# Ammonia Stress Disturbs Moult Signaling in Juvenile Swimming Crab *Portunus trituberculatus*

**DOI:** 10.3390/biology12030409

**Published:** 2023-03-06

**Authors:** Daixia Wang, Xiaochen Liu, Yan Shang, Xuee Yu, Baoquan Gao, Jianjian Lv, Jitao Li, Ping Liu, Jian Li, Xianliang Meng

**Affiliations:** 1 National Demonstration Center for Experimental Fisheries Science Education, Shanghai Ocean University, Shanghai 201306, China; 2 National Key Laboratory of Mariculture Biobreeding and Sustainable Goods, Yellow Sea Fisheries Research Institute, Chinese Academy of Fishery Sciences, Qingdao 266071, China; 3 Laboratory for Marine Fisheries Science and Food Production Processes, Qingdao National Laboratory for Marine Science and Technology, Qingdao 266071, China; 4 Key Laboratory of Aquatic Genomics, Ministry of Agriculture and Rural Affairs, Yellow Sea Fisheries Research Institute, Chinese Academy of Fishery Sciences, Qingdao 266071, China

**Keywords:** ammonia, swimming crab, MIH, moulting, moulting death syndrome

## Abstract

**Simple Summary:**

Ammonia is the most common contaminant in aquaculture systems, including intensive hatchery systems for crustaceans. Although it has been demonstrated that ammonia can result in abortive moulting and massive mortality of juvenile crustaceans, the underlying mechanisms are still unknown. The present work is aimed at examining the effect of ammonia exposure on the moulting process as well as molt signaling in the swimming crab *Portunus trituberculatus*, an important aquaculture species in China. We examined the survival rate and moulting rate of the juvenile crabs (C2) and analyzed the expression pattern of the genes in key components of molt signaling during a complete moulting cycle under different concentrations of ammonia. Our results revealed that ammonia has dose-dependent biphasic effects on moulting in juvenile swimming crab. Specifically, low levels of ammonia (5 mg/L) stimulated moulting, while high levels of ammonia (20 mg/L) suppressed the moulting process and caused moulting death syndrome (MDS). The gene expression analysis indicated that low levels of ammonia can reduce the expression of *MIH*, which encodes the key negative regulator of moulting, and trigger ecdysteroid biosynthesis and ecdysteroid signaling in the juvenile crabs. In contrast, though the high level of ammonia increased *MIH* expression, it still resulted in excessive ecdysteroids and over-activation of ecdysteroid signaling, which may contribute to the depressed moulting and MDS in the juvenile crabs. The novel findings of this study improve the understanding of ammonia toxicity in brachyura and provide valuable information for hatchery management of *P. trituberculatus*.

**Abstract:**

Ammonia is a significant concern during hatchery culture in brachyuran species, and its accumulation may lead to abortive moulting and large-scale deaths of the early juveniles. To date, the underlying mechanism for ammonia-induced alteration of the moulting process is still unknown. In this study, we aimed to investigate the effects of ammonia on the moulting as well as the potential mechanisms in early juveniles of the swimming crab *Portunus trituberculatus*, an important aquaculture species in China. We evaluated the survival rate and moulting rate of the juvenile crabs (C2) and analyzed the expression pattern of the genes in key components of molt signaling during a complete moulting cycle under different concentrations of ammonia nitrogen (the control group: <0.1 mg/L; the LA group: 5 mg/L; and the HA group: 20 mg/L). The results showed that: (1) the survival rate in the LA and HA groups was lower than that in the control group at the end of the experiment, and moulting death syndrome (MDS) was only observed in the HA group; (2) the moulting rate was higher in the LA group and lower in the HA group compared to the control group; (3) consistent with the results of the moulting experiment, *MIH* showed decreased expression, and genes related to ecdysteroid synthesis, ecdysteroid receptors, and responsive effectors exhibited increased expression in the LA group compared to the control group; and (4) although *MIH* expression was upregulated, increased expression of the genes associated with ecdysteroid synthesis, ecdysteroid receptors and downstream effectors still observed in the HA group. Our results indicated that low levels of ammonia can promote moulting in juvenile swimming crabs by inhibiting the expression of *MIH* and activating moult signaling, whereas high levels of ammonia inhibit moulting and lead to MDS through impairing moult signaling.

## 1. Introduction

The swimming crab *Portunus trituberculatus* is widely distributed in the coastal waters of Korea, Japan, China, and Southeast Asian countries [1]. This species is dominant in world Portunid fisheries and supports a large aquaculture industry in China. In 2020, its production reached 100,895 tons [2]. In recent years, with rising market demand and the expansion of swimming crab farming, the production of high-quality seed has been unable to meet the needs of the aquaculture industry, which has become an important limiting factor for the sustainable development of the industry. At the larval and juvenile crab stages, individuals are very sensitive to changes in environmental factors, such as temperature, ammonia, and nitrite [3,4]. Under high-density breeding conditions in indoor hatchery ponds, residual feeds and the excretion from the crabs may lead to a rapid accumulation of ammonia [5]. Studies have shown that ammonia is toxic to crustaceans and can affect the moulting process and even cause mass mortality due to failure of proper moulting, a phenomenon known as moulting death syndrome (MDS) [6,7]. Although most existing studies showed that ammonia affects the moulting process in crustaceans, its effects on moulting seem to be controversial in different species. Liao et al. [8] found that ammonia over 16.86 mg/L can cause a significant decrease in the moulting rate of juvenile *Portunus pelagicus*, and all juvenile crabs died from MDS when the concentration of ammonia nitrogen reached 134.88 mg/L. A study on the mud crab *Scylla serrata* found that the moulting rate was significantly reduced when the ammonia concentration was too high [9]. In the tiger crab *Orithyia sinica*, the molt interval was found to be shortened after ammonia exposure [10]. To date, the effects of ammonia exposure on the moulting of *P. trituberculatus* are still unknown.

In crustaceans, moulting, the process of shedding the old exoskeleton and synthesising a new one, is required for somatic growth. This process is coordinated by a complex interplay of hormones and downstream signaling [11]. Molt-inhibiting hormone (MIH), a neuropeptide from the X-organ sinus glands (XO-SG) complex in the eyestalk, can suppress the synthesis/secretion of ecdysteroids from the Y-organs (YO) [12,13,14]. Ecdysteroids, synthesized from cholesterol through Halloween genes, are moulting hormones in crustaceans. These hormones can bind to the heterodimer receptor complex composed of the ecdysteroid receptor (EcR) and the retinoid-X receptor (RXR) [15,16,17,18]. The binding can activate the transcription of early responsive genes (e.g., the ecdysone-induced protein 75 gene (E75) and nuclear hormone receptor 3 (HR3)), and then the transcription of late responsive genes (e.g., chitinase (Chi)) that are responsible for the moulting procession [18,19,20,21]. 

It has been reported that numerous environmental factors, such as temperature, salinity, and photoperiod, have been shown to disrupt moult signaling steps and thus affect the moulting process in crustaceans [22,23,24]. For example, low temperature at 14 °C leads to a subdued *EcR* level and prevents moulting in early juvenile mud crab *Scylla paramamosain*. In contrast, high temperature at 32 °C, induces a significant increase in *EcR* expression and dramatically reduced moulting interval in juvenile crabs [25]. A low salinity of 10 psu increases *EcR* expression and significantly shortens the moulting interval, while a high salinity of 40 psu results in a decreased level of *EcR* expression and delayed moulting in *Macrophthalmus Japonicus* [26]. Under low salinity stress, the expression level of *Chi* is reduced significantly, and moulting frequency concomitantly decreases in *Litopenaeus vannamei* [27]. There has been no report regarding how environmental ammonia interferes with moult signaling in the swimming crab.

The present study aimed to investigate the effects of ammonia exposure on moulting in early juvenile *P. trituberculatus* and explore the underlying mechanisms. Here, we assessed the survival and the moulting process of juvenile swimming crabs during a complete moulting cycle under different concentrations of ammonia nitrogen (<0.1 mg/L, 5 mg/L, and 20 mg/L). In addition, we also analyzed the expression pattern of *MIH* and the genes related to ecdysteroid synthesis (shadow (*Sad*), spook (*Spo*), and disembodied (*Dib*)), ecdysteroid receptors (*EcR* and *RXR*), and downstream responsive genes (*E75*, *HR3*, and *Chi*) in different groups. The results of this study can provide a better understanding of ammonia-induced abortive moulting in crustaceans, and useful data for improving hatchery management of swimming crabs.

## 2. Materials and Methods

### 2.1. Experimental Animals

Because ammonia usually accumulates in indoor ponds over time and lead to mass mortality at the late stage of hatchery, the second stage juvenile swimming crabs (C2), which are in their last moulting cycle before being released to outdoor culture ponds, were chosen as the experimental animals in this study. The juvenile *P. trituberculatus* were obtained from Haifeng Company (Weifang, China). Newly metamorphosed C1 juvenile crabs (7.9 ± 0.6 mg) were transferred from indoor hatchery ponds to experimental tanks (40 L) and acclimated at optimum conditions for three days, where aeration was provided continuously, temperature was maintained at 26 °C ± 0.5 °C, salinity was 30.3 ± 0.3, ammonia nitrogen concentration was below 0.1 mg/L, the pH was 7.6 ± 0.2, and photoperiod was set as 12 h of light: and 12 h of dark. Seawater temperature, salinity, and pH were measured using a YSI Professional Plus multi-parameter water quality meter (Yellow Springs Instrument Co., Inc., Yellow Springs, OH, USA). Ammonia nitrogen concentration was determined using the salicylic acid method with a spectrophotometer. One third of the rearing water was exchanged every day using fresh, equivalent-temperature seawater. The juvenile swimming crabs were fed with adult *Artemia ad libitum* every 8 h, and the leftover feed was removed before feeding. After the three day acclimation, most of the C1 crabs moulted into C2 individuals, and the newly moulted C2 crabs were used for subsequent experiments.

### 2.2. Survival and Moulting Experiment

Our preliminary experiments found that ammonia exposure may have biphasic effects on juvenile crabs moulting. Therefore, we chose two typical exposure concentrations that have opposite effects on moulting. This study was conducted at three ammonia nitrogen concentrations, including natural seawater (below 0.1 mg/L, the control group), 5 mg/L (the LA group), and 20 mg/L (the HA group). The ammonia nitrogen concentration in the different experimental groups was adjusted with 1 M ammonium chloride (NH_4_Cl) stock solution, and the culture conditions were the same as those in the acclimation period. A total of 180 newly molted C2 crabs were equally and randomly allocated into the three groups, and there were three replicates for each experimental group. The number of surviving crabs and those that moulted successfully were recorded every 12 h during the whole moulting cycle from C2 to C3. In addition, the stages at which the individuals died and whether they died from MDS were also recorded.

### 2.3. Sample Collection for Gene Expression Analysis

To explore the potential mechanisms for ammonia-induced abortive moulting, crabs from different groups were collected to analyze the expression pattern of genes related to the moulting process during the moulting cycle from C2 to C3. A total of 900 newly moulted C2 juveniles were equally allocated into the three groups, and each group had three replicates. The culture conditions were the same as those during the acclimation period. For each group, 30 crabs were randomly sampled at 24 h, 48 h, 72 h, 96 h, and 120 h after ammonia exposure. The eyestalk and the remaining parts of each crab were collected, immediately frozen in liquid nitrogen, and stored at −80 °C for the subsequent gene expression analysis.

### 2.4. Gene Expression Analysis

Since the eyestalks of the juvenile crabs used in this study are too small to extract enough RNA, the eyestalks of ten individuals were pooled together as one replicate, there were three replicates for each group at the same sample point. Similarly, the somatic parts of ten crabs were also pooled for RNA extraction. The total RNA of the samples was isolated using the TransZol Up Plus RNA Kit (TransGen Biotech, Beijing, China), following the manufacturer’s protocol. RNA integrity was assessed using agarose gel electrophoresis (Bio-Rad, Hercules, CA, USA), and RNA quantity was determined using Nanodrop (Thermo Fisher Scientific, Waltham, MA, USA). Reverse transcription of the total RNA into complementary DNA (cDNA) was carried out using the Evo M-MLV RT mix kit with gDNA Clean for qPCR (Accurate Biology, Changsha, China).

Quantitative real-time PCR (qPCR) was performed to analyze the expression of *MIH* and the genes associated with ecdysteroid synthesis (*Sad*, *Spo*, and *Dib*), ecdysteroid receptors (*EcR* and *RXR*), and downstream responsive genes (*E75*, *HR3*, and *Chi*), using the SYBR^®^ Green Pro Taq HS Premix qPCR Kit II (Accurate Biology, China) in the ABI 7500 fast qPCR system (Applied Biosystems, Foster City, CA, USA). The reaction system was 10 μL, containing 5.0 μL 2× SYBR Green Pro Taq HS Premix II, 0.2 μL ROX Reference Dye, 0.4 μL forward and reverse primers, 3.0 μL RNA-free water, and 1.0 μL cDNA template. The PCR reaction conditions were set as follows: initial denaturation and enzyme activation at 95 °C for 30 s, followed by 40 cycles of 95 °C for 5 s and 60 °C for 30 s. The relative expression of moulting-related genes was calculated using the 2^−ΔΔCT^ method [28]. The relative expression of genes was normalized to *β-actin* [29,30] and the fold-change from the control group CT value was calculated. The specific primers used in this study are listed in Table 1.

### 2.5. Statistical Analyses

The Mantel–Cox test was used to compare the survival curves of the LA and HA groups with respect to the control group. The moulting rate and gene expression among different groups at each time point were analyzed with one-way analysis of variance (one-way ANOVA) after checking for normality and homogeneity of variance in the data. If significant differences were found, Duncan’s post-hoc test was used to identify the differences between the treatments. The correlation between the expression of all the tested genes was performed using Pearson correlation analysis. The statistically significant level was set as *p* < 0.05.

## 3. Results

### 3.1. Survival and Molting

**Figure 1:**
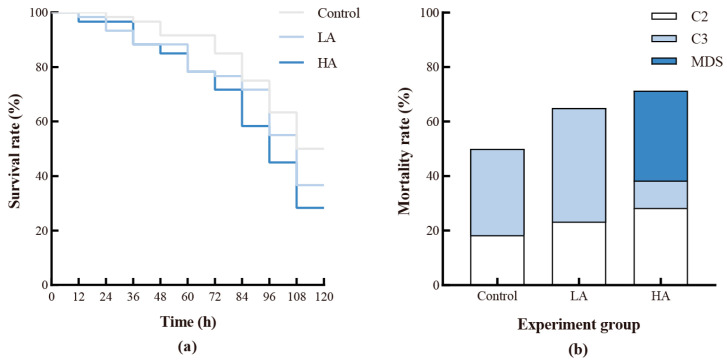
Survival rate (**a**) and mortality composition (**b**) of *P. trituberculatus* in different groups.

Survival analysis using the Mantel–Cox test showed that ammonia exposure significantly reduced the survival of the juvenile crabs during the moulting cycle from C2 to C3 (*p* < 0.05) (Figure 1a). As shown in Figure 1b, over half of the deaths of the juveniles in the control and LA groups occurred after successful moulting, while only 10% died after moulting to C3, and 33% died from MDS in the HA group.

**Figure 2:**
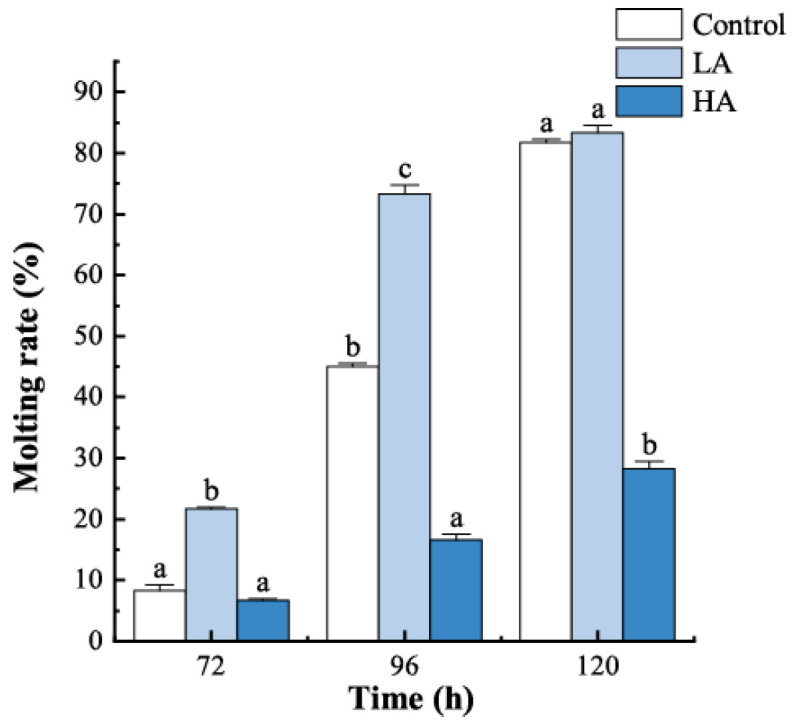
Molting rate of juvenile *P. trituberculatus* at different ammonia nitrogen concentrations. At the same time, the letters indicate significant differences between experimental groups (*p* < 0.05).

The juvenile crabs in all three groups started moulting after 72 h, and all of the surviving crabs completed moulting before 120 h (Figure 2). Ammonia exposure significantly affected the moulting rate at different times (72 h, *p* = 0.020; 96 h, *p* = 0.001; and 120 h, *p* = 0.000). At 72 h, the moulting rate in the LA group was significantly higher than that in the other groups, which were 2.60 and 3.25 times that of the control and HA groups, respectively. At 96 h, the highest moulting rate was also observed in the LA group. The moulting rate in the HA group was significantly lower than the other two groups at 96 h and 120 h.

### 3.2. MIH Expression

**Figure 3:**
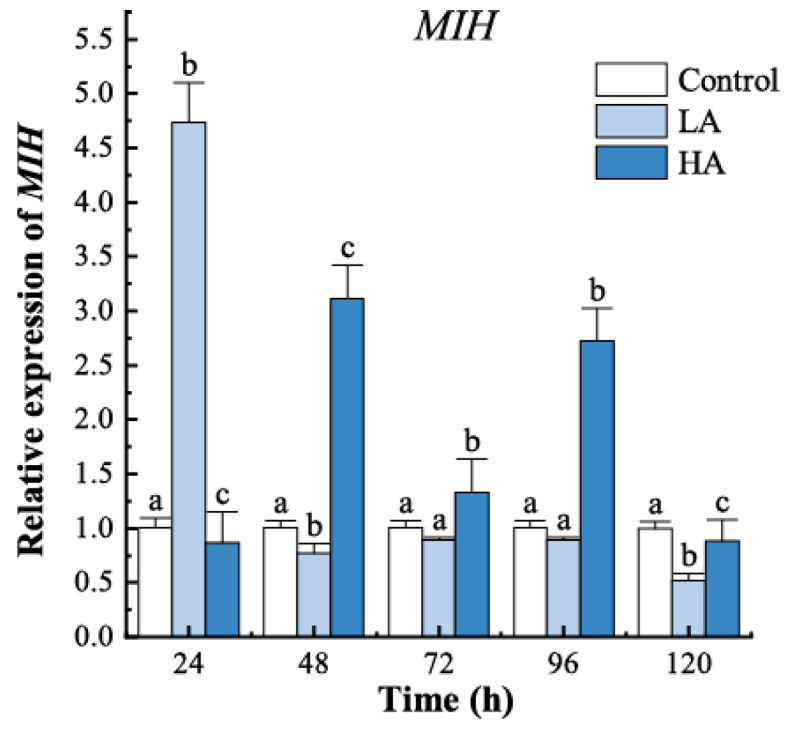
*MIH* mRNA expression in juvenile crabs exposed to ammonia nitrogen stress. At the same time, the letters indicate significant differences between experimental groups (*p* < 0.05).

The expression of *MIH* in the LA group was significantly higher than the control level only at 24 h (*p* < 0.05) (Figure 3) and then remained consistently lower than the control group from 48 h to 120 h. The HA group exhibited higher *MIH* expression than the other two groups from 48 h to 96 h.

### 3.3. Expression of the Halloween Genes

**Figure 4:**
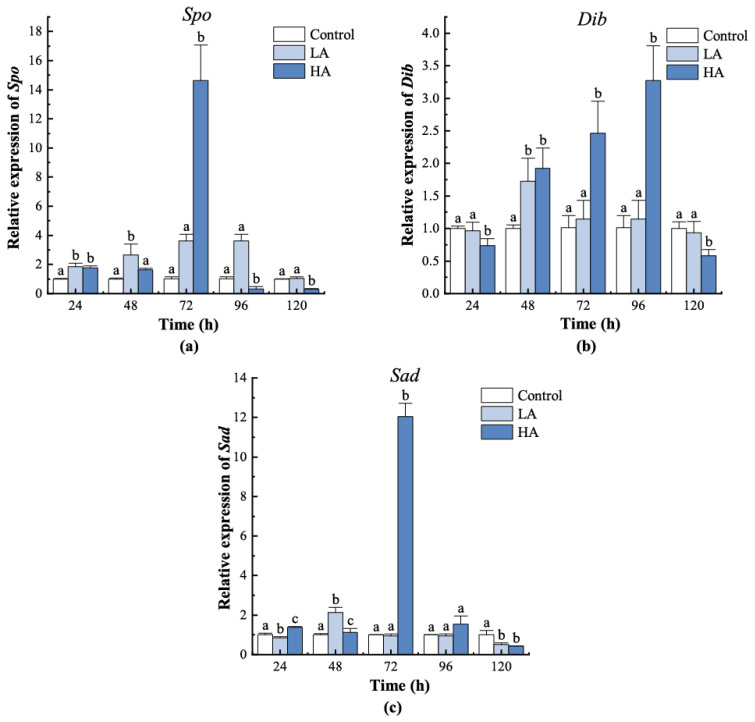
Expression patterns of Halloween family genes in juvenile crabs exposed to ammonia nitrogen stress. (**a**) *Spo*, (**b**) *Dib*, and (**c**) *Sad*. At the same time, the letters indicate significant differences between experimental groups (*p* < 0.05).

The expression of the Halloween genes was significantly affected by ammonia stress (Figure 4). In the LA group, the expression of *Spo* was higher than that of the control from 24 h to 96 h after exposure (*p* < 0.05) and returned to the control level at 120 h (*p* > 0.05). Both *Dib* and *Sad* in the LA group were significantly upregulated at 48 h and then decreased gradually. *Spo* in the HA group shared a similar expression pattern with that in the LA group, whereas *Dib* initially showed a lower expression than the control and LA groups but then exhibited a higher expression from 48 h to 96 h, compared with the control. *Sad* expression in the HA group was slightly upregulated at 24 h and 48 h and dramatically increased at 72 h. All three Halloween genes showed higher expression in the HA group than in the other groups at the initiation of moulting (72 h, *p* < 0.05).

### 3.4. Expression of Ecdysteroid Receptors

**Figure 5:**
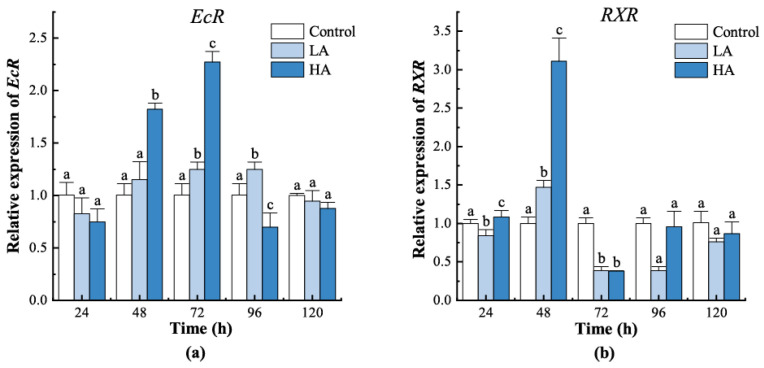
Expression patterns of receptors in the moulting signal pathway from juvenile crabs under ammonia nitrogen stress. (**a**) *EcR*, (**b**) *RXR*. At the same time, the letters indicate significant differences between experimental groups (*p* < 0.05).

For the LA group, the mRNA level of *EcR* was significantly upregulated at 72 h and 96 h (*p* < 0.05) (Figure 5) and returned to the control level at 120 h (*p* > 0.05). For the HA group, *EcR* mRNA levels were upregulated at 48 h and 72 h which are higher than the control group (*p* < 0.05), while they were downregulated at 96 h. *RXR* expression in the LA group showed lower expression than the control group at most sample points except 48 h. *RXR* levels in the HA group were initially upregulated at 24 h and 48 h (*p* < 0.05), became downregulated at 72 h (*p* < 0.05), and then returned to the control level at 96 h and 120 h (*p* > 0.05).

### 3.5. Expression of Ecdysteroid-Responsive Genes 

**Figure 6:**
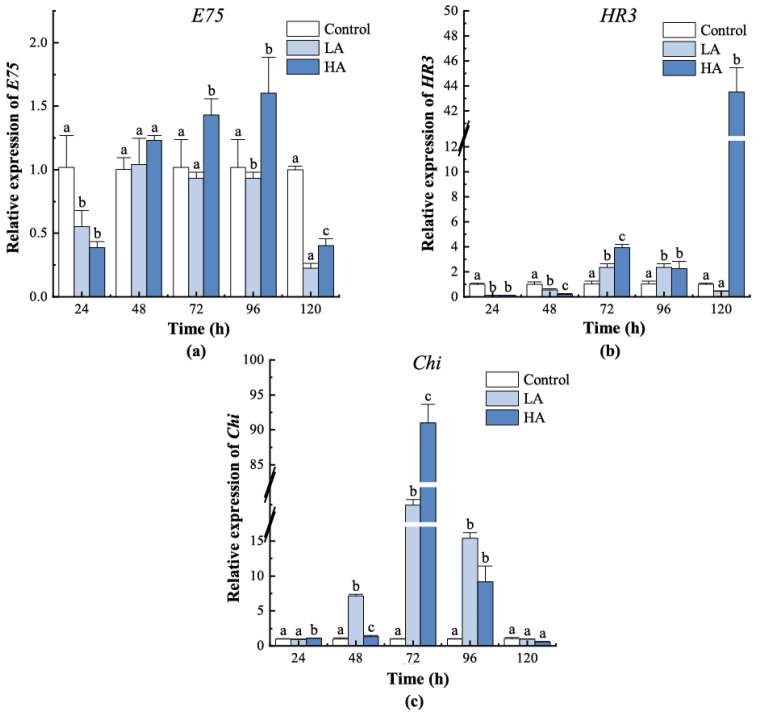
Gene expression pattern of downstream regulators in juvenile crabs exposed to ammonia nitrogen stress. (**a**) *E75*, (**b**) *HR3*, and (**c**) *Chi*. At the same time, the letters indicate significant differences between experimental groups (*p* < 0.05).

*E75* in both the LA and HA groups downregulated at 24 h, and its expression in the LA group remained at levels similar to the control group from 48 h to 96 h, while the expression in the HA group was significantly higher than that in the control group (*p* < 0.05) (Figure 6). *Chi* in the LA and HA groups shared a similar expression pattern and showed a significant increase in expression in both groups from 48 h to 96 h (*p* < 0.05). *HR3* expression pattern in the LA and HA groups was also similar before 96 h, but its expression in the HA group increased greatly at 120 h which was significantly higher than those in the control and LA groups (*p* < 0.05). All three ecdysteroid responsive genes exhibited higher expression in the HA group than the other two groups at the initiation of moulting (72 h, *p* < 0.05).

### 3.6. Correlation between the Expression of All the Detected Genes

**Figure 7:**
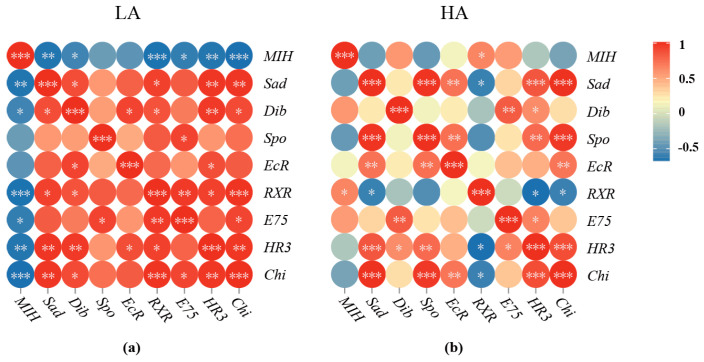
Correlation analysis of the moulting signals in the juvenile crabs under ammonia nitrogen stress. (**a**) correlation analysis for the LA group; (**b**) correlation analysis for the HA group. * represents a significant difference between two genes (*p* < 0.05), ** represents *p* < 0.01, and *** represents *p* < 0.001.

A significantly negative correlation of gene expression was observed between *MIH* and the genes related to ecdysteroid synthesis (*Sad*, *p* < 0.01; *Dib*, *p* < 0.05), ecdysteroid receptors (*RXR*, *p* < 0.001), and downstream responsive genes (*E75*, *p* < 0.05; *HR3*, *p* < 0.01; *Chi*, *p* < 0.001) in the LA group (Figure 7a). In contrast, *MIH* only showed a positive correlation in expression with *RXR* (*p* < 0.05), while exhibiting no significant correlation with the other tested genes (*p* > 0.05) (Figure 7b).

## 4. Discussion

Ammonia is the most common environmental pollutant in aquaculture systems; in fact, it is the main limiting factor in aquaculture practice [32]. Many studies have shown that ammonia is toxic to aquatic animals, including crustaceans, and it affects survival and the moulting process in decapod species [4,33]. Almost all previous studies reported that ammonia reduces survival in decapod species; however, its effects on moulting appear to be opposite in different species [8,9,34]. For example, ammonia exposure at 16 mg/L inhibits the moulting of the mud crab *S. paramamosain* and causes the death of all individuals at 128 mg/L from MDS [22]. In contrast, ammonia at 50–150 mg/L can significantly reduce the intermoult period and increase moulting frequency in the tiger crab *O. sinica* [10]. The results of this study showed that the moulting rate was significantly higher in the LA (5 mg/L) group and lower in the HA (20 mg/L) group, compared with the control group. It is noteworthy that none of the individuals in the LA group died from MDS, while 33% of the crabs in the HA group died of MDS. These results for the first time indicated that environmental ammonia has dose-dependent, biphasic effects on the moulting of juvenile swimming crabs. Specifically, low levels of ammonia stimulate the moulting process, while high levels of ammonia suppress the moulting process and cause moulting failure. 

The moulting of crustaceans is controlled by a complex signaling network involving multiple hormones and their downstream signaling pathways [35]. To explore the underlying mechanisms for the ammonia-induced alteration of the moulting process, we analyzed the expression of nine genes implicated in key components of molt signaling in juvenile *P. trituberculatus* during a complete moulting cycle under ammonia stress. MIH is considered to be the primary neurohormone responsible for crustacean moulting inhibition, and it works by negatively regulating ecdysteroids biosynthesis [36,37,38,39]. Recent studies have reported that injection of recombinant MIH represses expression of the Halloween genes, which are essential for ecdysteroidogenesis, and catalyzes the conversion of cholesterol to ecdysteroids. Conversely, withdrawal of MIH by eyestalk ablation or knockdown of *MIH* by RNAi induces expression of the Halloween genes [17,20,40,41,42]. These results indicated that the inhibitory effect of MIH on ecdysteroids biosynthesis occurs via transcriptional repression of the Halloween genes in YO [14,20]. In the present study, *MIH* was downregulated, and accordingly, the Halloween genes, namely *Spo*, *Dib,* and *Sad*, were upregulated in the LA group. In addition, Pearson’s analysis showed that *MIH* exhibited a significantly negative correlation in expression with *Dib* and *Sad*. The results were consistent with the moulting experiment, which showed a higher moulting rate in the LA group at the early stage after ammonia exposure, suggesting that ammonia at 5 mg/L promotes moulting in juvenile swimming crabs by inhibiting *MIH* expression, which in turn induces expression of the Halloween genes and stimulates ecdysteroidogenesis.

Considering that the HA group had a lower moulting rate compared to the control group, we expected increased expression of *MIH* and decreased expression of the Halloween genes. Unexpectedly, upregulation of both *MIH* and the tested Halloween genes was observed in the HA group, indicating that environmental ammonia at 20 mg/L can abolish the inhibitory activity of MIH on the transcription of the Halloween genes in juvenile *P. trituberculatus*. However, to date, the signaling mechanisms by which MIH regulates the Halloween genes are not well understood [43], and the MIH receptor remains unidentified [44,45]. Several studies in decapods have suggested that the receptors for MIH are GPCRs located in YO and that the signal is transduced by cyclic nucleotide second messengers [45]. It is therefore possible that high levels of ammonia may disrupt the membrane receptor-mediated signaling of MIH, thereby promoting ecdysteroid biosynthesis. 

In crustaceans, ecdysteroid action is mediated via the heterodimerization of two nuclear receptors, EcR and its partner, RXR [46]. The EcR/RXR heterodimer binds to hormone response elements to induce the expression of early genes, and these early responsive gene products are transcription factors that induce ecdysteroid signaling cascade [47,48]. A number of studies in decapods such as *Gecarcinus lateralis* have demonstrated that *EcR* expression is generally in accordance with the titer of ecdysteroid in hemolymph during a moult cycle and that ecdysteroid injection upregulates expression of *EcR*, indicating that ecdysteroid triggers *EcR* expression [49,50,51]. We found that the levels of *EcR* were increased at both concentrations of 5 mg/L and 20 mg/L in this study. Furthermore, the early ecdysteroid-responsive genes, *E75* and *HR3*, and the terminal gene of ecdysteroid signaling, *Chi*, also exhibited upregulated expression after ammonia exposure. Taken together, the results further indicated that ammonia stress can induce ecdysteroidogenesis and activate ecdysteroid downstream signaling in the juvenile swimming crabs. 

Contrary to the results of the present study, our recent study in adult swimming crabs found that ammonia stress results in a significant reduction of *EcR* [52]. Similarly, Si et al. [53] found that expression of ecdysteroid responsive genes, *Chi,* and ecdysteroid-regulated-like protein, are significantly downregulated after ammonia exposure. These results suggested that the effects of ammonia on ecdysteroid signaling in the swimming crab are developmental stage specific. It is known that ecdysteroids display pleiotropic functions at different stages of the life cycle in crustaceans, and they orchestrate metamorphosis and moulting at larval and growing stages, while regulating vitellogenesis at reproductive-developmental stages [24,52]. The pleiotropic activity of ecdysteroid and the developmental stage-specific effects of ammonia on ecdysteroid signaling may lead to diverse consequences following ammonia exposure, and thus more work that focuses on the individuals at different developmental stages should be carried out to clarify the adverse effects of ammonia in the swimming crabs and the potential mechanisms.

*EcR* expression has been extensively used as a biomarker for accessing disruption of ecdysteroid signaling as well as the moulting-interfering effects of many environmental stressors [25,51,54] and toxicants [55], because it plays important roles in the ecdysteroid signaling pathway and is very sensitive to environmental changes [56]. In this study, expression of *EcR* and ecdysteroid-responsive genes in both ammonia-treated groups were significantly upregulated, confirming that *EcR* could be a reliable biomarker for monitoring ecdysteroid signaling in juvenile swimming crab under ammonia stress. However, *EcR* expression seems not to be a suitable biomarker for evaluating the moulting-interfering effect of ammonia, as moulting was induced and suppressed in the LA and HA groups, respectively, though expression of *EcR* in these two groups showed a similar trend after ammonia exposure.

The precise regulation of ecdysteroid levels is considered fundamental to successful moulting of crustaceans [25,56]. Previous studies showed that an injection of ecdysteroids at a low dose can accelerate moulting, but administration at a high dose may cause failure of moulting and an increase in mortality [57,58,59]. In this study, though most of the tested genes showed similar trends in the LA and HA groups, there was an obvious difference in the moulting rate and the number of individuals who died from MDS between the two groups. The difference may be associated with the levels of ecdysteroid in these groups under ammonia stress. Previous studies reported that the expression of the Halloween genes responsible for ecdysteroid biosynthesis usually showed a positive correlation with ecdysteroid levels [60]. The higher expression of all the tested Halloween genes in the HA group, particularly at the initiation of the moulting (72 h), may lead to a higher level of ecdysteroids compared with the LA group. This is supported by the higher expression of *EcR* and the ecdysteroid responsive genes in the HA group, as the expression of these genes is reported to be induced by ecdysteroid in a dose-dependent manner [41,50]. Thus, we propose that a high level of ammonia could result in excessive ecdysteroids and over-activation of ecdysteroid signaling in juvenile *P. trituberculatus*, thereby causing depression of the moulting process and death due to moulting failure. Because of the difficulty in getting hemolymph from the early juveniles, we were not able to analyze the levels of ecdysteroids in this study. More detailed studies that accurately determine the ecdysteroid levels in juvenile individuals are still required to confirm this hypothesis.

In summary, to our knowledge, this is the first study to investigate the effects of ammonia on moulting and molt signaling in juvenile *P. trituberculatus*, an important aquaculture species in China. The results revealed that ammonia led to a decreased survival rate in both ammonia-exposed groups. Given that even a low level of ammonia exposure (5 mg/L) can significantly reduce the survival of the juvenile crabs, particular attention should be paid to ammonia control during the intensive hatchery of *P. trituberculatus*. In addition, we found ammonia had dose-dependent biphasic effects on juvenile swimming crab moulting. Low concentrations of ammonia (5 mg/L) promoted moulting, while high concentrations of ammonia (20 mg/L) resulted in a decreased moulting rate and caused MDS. The gene expression analysis indicated that low levels of ammonia can reduce *MIH* expression, and trigger ecdysteroid biosynthesis and ecdysteroid signaling in the juvenile crabs. In contrast, while high levels of ammonia increased *MIH* expression it also resulted in excessive ecdysteroids and over-activation of ecdysteroid signaling, which may contribute to the depressed moulting and MDS in juvenile crabs. The findings of this study improve our understanding of the adverse effects of ammonia stress on juvenile *P. trituberculatus* and the underlying mechanisms, which provides useful information for aquaculture management during hatching. 

## Figures and Tables

**Table 1:** Primer sequence used in the research.

Gene	Forward Primer	Reverse Primer
*β-actin*	CGAAACCTTCAACACTCCCG	GGGACAGTGTGTGAAACGCC
*MIH*	CCGCTGAATCTCACACCGAT	AAGGTTCCGCTGAGTTCCTG
*E75* ^1^	CGAGAGCCTAGTGATGTA	ATGAGTGATGAGCGAGTA
*HR3*	CTCACGAGGAGCTCTGGTTC	TGCGAGAATTTCCTGAATCC
*EcR* ^1^	TAAGTGATGACGACTCGGATGC	ACGAGCAAGCCTTTAGCAGTG
*RXR* ^1^	AGCGTCAGAGGACAAAAGGC	TGGTCCAGTGGCTGCTCAT
*Chi* ^1^	CCCAGCCGATAGGAAGACC	CGCTGTCAGTATCATTCCGTTAG
*Sad* ^1^	CACGGCATTTTCAAGGAGA	AAGGCGTCATCCAGGCACT
*Dib* ^1^	TGCGAGTCTGCTTGAGGTG	AGCCATTGTCAGTGGGGAG
*Spo* ^1^	GGGACGAGCCCAATAAGTT	CTGGTGCTGAAAGGGATGA

^1^ The primers for *E75*, *EcR*, *RXR*, *Chi*, *Sad*, *Dib,* and *Spo* were from Xie et al. (2016) [31] and Xie et al. (2016) [20].

## Data Availability

All data generated by this study are available in this manuscript.
